# The Kaplan Meier estimates of mortality and its predictors among newborns admitted with low birth weight at public hospitals in Ethiopia

**DOI:** 10.1371/journal.pone.0238629

**Published:** 2020-09-11

**Authors:** Samuel Dessu, Aklilu Habte, Molalegn Mesele

**Affiliations:** 1 Department of Public Health, College of Medicine and Health Sciences, Wolkite University, Wolkite, Ethiopia; 2 Department of Public Health, College of Medicine and Health Sciences, Wachamo University, Hossana, Ethiopia; 3 Department of Midwifery, College of Medicine and Health Sciences, Wolaita Soddo University, Wolaita Soddo, Ethiopia; ESIC Medical College & PGIMSR, INDIA

## Abstract

**Background:**

Globally, every year greater than twenty million newborns are born weighing less than 2500grams, of which over 96% of them were in developing countries. It contributes to sixty to eighty percent of all neonatal deaths, annually. These infants weighing less than 2500gram will have a poor immune system and are at risk of morbidity and mortality. This study was aimed to investigate the survival status and predictors of mortality among neonates admitted with low birth weight at public hospitals in Ethiopia.

**Method:**

A prospective cohort study was conducted among selected 216 newborns admitted with low birth weight who were admitted in neonatal intensive care unit at Arba Minch General hospital, Sawla General Hospital and Chencha district hospital from 1^st^ March 2018 to 28^st^ February 2019. Data were entered into Epi data version 3.02 and exported to STATA V 14 for analysis. The Kaplan Meier survival curve together with log rank test was used to estimate the survival time of the newborns. Variables which had p-value <0.05 in multivariable analysis using the cox proportional hazard model were declared as statistically significant.

**Result:**

The cumulative proportion of surviving at the end of third days was 99.01% (95%CI: 96.11, 99.75). Similarly it was 97.81% (95%CI: 94.25, 99.18), 96.27% (95%CI: 91.76, 98.33) and 94.33% (95%CI: 88.72, 97.19) at the end of fourth, fifth and sixth day respectively. The overall mean survival time was 17.13 days (95%CI: 12.76, 21.49). The incidence of mortality among neonates admitted with low birth weight was 83 per 1000 live births. Mothers with DM (AHR:4.79; 95%CI:1.15, 19.8), maternal HIV infection(AHR:6.47;95%CI:1.43,29.3), not keeping the newborn under KMC(AHR:13.0;95%CI:3.42, 49.5) and initiating exclusive breast feeding within one hour(AHR:0.19;95%CI:0.04, 0.95) were statistically significant in multivariable cox regression analysis.

**Conclusion:**

The risk of mortality among neonates admitted with low birth weight was high at the early admission period and the incidence of mortality was high. Maternal history of diabetes mellitus, HIV/AIDS infection, keeping the newborn under kangaroo mother care and exclusive breast feeding initiation were statistically significant predictors of mortality. Special emphasis should be given for neonates with low birth weight at the early follow up periods and Kangaroo mother care practice should be a usual practice.

## Background

Low birth weight (LBW) is defined by the World Health Organization (WHO) as newborn weight less than 2500 grams [1]. It shows the public health concern, maternal health condition and nutritional status across the area. It continues to be related with a wide range of both short term and long-term outcomes if not early screened and treatment initiated [2, 3]. Certain disparities on the survival status of the child were observed across the globe in certain time frames [4].

Globally, every year greater than twenty million newborns have LBW, of which over 96% of them were in developing countries. These LBW infants will have a poor immune system and at are at a higher risk of early growth retardation, nutritional deficiencies, decreased muscle strength throughout the life span, higher occurrence of cardiovascular disease, mental disabilities, infectious diseases, delay in developmental status, lower intelligence quotient (IQ) which interferes their school and work performance and death during infancy and childhood ages [5–7]. It is one of the leading and foremost indirect causes of deaths among neonates [8]. It contributes to sixty to eighty percent of all neonatal deaths, annually [9].

World health organization (WHO) launches a strategy mentioned as Global Nutrition Targets 2025: LBW policy brief to achieve a thirty percent reduction in the number of LBW newborn by the year 2025. This strategy will contribute to a three percent reduction per year between the time frame 2012 and 2025 and lowers nearly twenty million to fourteen million infants with low weight at birth [1]. This policy will be achieved through intensive follow up of the neonates admitted with low birth weight and appropriate health interventions through determining the time that neonate will die.

The Ethiopian demographic health survey (EDHS 16) indicate that the estimated neonatal mortality rate (NMR) at 2015/16 was twenty eight per 1000 live births and this estimate accounts 47.5% of the total rate of child mortality [10, 11]. From these, LBW accounts majority of the estimate. The risk of mortality among newborns admitted with LBW in Ethiopia was different in terms of certain factors such as socio-demographic factors such as age, place of residence, marital status, maternal educational status, and family monthly income, neonatal characteristics, maternal medical and obstetrical factors [12, 13]. Studies conducted at different regions of Ethiopia declared that the NMR is incomparably high [12–14].

Despite the magnitude of low birth weight, the survival time of neonates, incidence of mortality among neonates admitted with low birth weight was not well investigated. Therefore; this study was aimed to estimate the survival and the mortality and investigate predictors of mortality of low birth weight neonates admitted at public hospitals in Ethiopia, 2018–2019.

## Methods

### Study setting, period and design

A prospective cohort study was conducted at neonatal intensive care units of Arba Minch General Hospital, Chencha District Hospital and Sawla General Hospital from 1^st^ March 2018 to 28^th^ February 2019.

### Populations

All neonates admitted to the neonatal intensive care unit (NICU) with diagnosed low birth weight and who had age less than 28 days were the source populations. The follow up period was initiated at admission to the NICU on 1^st^ March 2018 and closed on 28^th^ February 2019. The neonates were followed until they had a maximum age of 28 days. The end point of this study was either death or recovery, loss to follow up, transferred to another institution and follow up time was completed without outcome happening. The study was an open cohort study and any newborn with low birth weight within the study period was entered and leaves the study. Within this time frame, there were a total of 216 neonates with diagnosed low birth weight and the study was conducted among all the diagnosed cases.

### Variables of the study

The dependent variable was time to death and the independent variables were socio-demographic factors (age of the neonate, sex of the neonate, maternal age, marital status of the mother, religious status, educational status of the mother, maternal occupational status, family size, estimated monthly income, residence, distance between home and hospital, maternal habit of alcohol intake and maternal habit of khat chewing), maternal medical and obstetrical factors (ANC visit, gravidity, parity, place of delivery, delivery attendant, gestational age, history of maternal DM, HIV/AIDS, iron deficiency anemia, pregnancy induced hypertension, UTI/STI, intra-partum fever, history of infant death and mode of delivery) and neonatal factors (Cry immediately at birth, first minute APGAR score, fifth minute APGAR score, resuscitation at birth, exclusive breast feeding initiation, feeding habit of the newborn, kept in kangaroo mother care within one hour and concurrent health problem).

### Operational definition

#### Maternal iron deficiency anemia

The hemoglobin level of a postnatal mother less than 11gm/dl.

#### Maternal history of sexually transmitted diseases

If she has chlamydia OR human papilloma virus OR syphilis OR Gonorrhea OR trichomoniasis OR herpes.

#### Habit of chewing khat

Individuals who were chewing khat within 3 months preceding the study.

#### Gravidity

The total number of times that the woman has been pregnant.

#### Concurrent neonatal health problem

A low birth weight neonate who had perinatal asphyxia OR birth injury OR hypothermia OR clinical or confirmed sepsis OR malaria OR neonatal jaundice OR any other health problem.

### Data collection tool, procedure and quality control measures

Data were collected by trained data collectors using a structured checklist. Both primary and secondary data were used. Primary data were collected from the mothers after admission to the hospital (post admission) at the postnatal ward, which was private for each of the mothers. Data quality was assured by caring out careful design of data extraction tools, training of both the data collectors and supervisors. Over more; pretest was conducted in 5% of the populations to improve the skill of data collectors and to ensure the consistency of the data extraction tool.

### Data processing and analysis

Epi data version 3.02 was used to enter the data, code the data, edit the data and clean the data and finally exported to STATA version 14 to conduct statistical analysis. The empirical relationship among the outcome variable and the explanatory variables was determined using bivariate statistical analysis. Both Crude hazard ratio (CHR) and adjusted hazard ratio (AHR) together with the corresponding 95% confidence interval and P-value were used to assess the strength of association and statistical significance. The Kaplan Meier survival curve together with log rank test was fitted to determine the survival time. Variables which had p-value <0.05 in bivariate analysis were considered as candidate for multivariable analysis and variables which had p-value <0.05 in multivariable cox regression analysis were considered as statistically significant.

### Ethical consideration

Ethical clearance was obtained from Arba Minch University, college of medicine and health sciences ethical review board. In addition; permission letter was obtained from the Arba Minch University. A written consent was obtained directly from the mothers. Mothers who did not read and write were informed about the information written on the informed consent. Over more, mothers were informed about the objective and significance of the study prior to the data collection. Appropriate measures were applied to ensure the confidentiality of the data.

## Results

### Socio demographic characteristics

In this study, a total of 216 neonates admitted with low birth weight were involved. Among them, 18 neonates were died. A total of 175(81%) of the neonates were categorized under the age less than seven days. This age category accounts 11(61.1%) of them were of the dyed neonates. In addition; a total of 133, 10, nine and seven neonates were admitted at the age of first, second, third and fourth days of life respectively. There was no any observed death at the first days of life while equal numbers of neonates (two neonates) were died at second, third and fourth days of life respectively. Males account 12(65.7%) of the total admitted newborns with low birth weight and 61.1% of the dyed neonates. In considering maternal age, 29(13.4%), 147(68.1%) and 40(18.5%) were categorized under less than 20 years old, 20–34 years old and more than 34 years old respectively. Similarly, 3(16.7%), 6(33.3%) and 9(50%) of the dyed new born categorized on the same maternal age respectively.

In considering the marital status of the mothers, nearly one tenth (9.7%) of the mothers were singles in their marital status and 77.8% of the dyed neonates were delivered from single mothers. Regarding their religious status, majority of the mothers were Orthodox and Protestant which accounts 94(43.5%) and 70(32.4%) respectively. Most of the mothers (30.6%) attended grade 1–8 followed by read and write (24.1%). The remaining 28(13%), 44(20.4%) and 26(12%) were unable to read and write, grade 9–12 and college and above in their study.

Regarding the occupational status of the mothers, nearly one tenth (10.2%) and one fourth (25.5%) of the mothers were house wives and self-employee respectively. The remaining 31%, 22.7% and 10.6% were farmers, merchants and civil servants respectively. In considering the family size and estimated monthly income of the families, 101(46.8%) have family size less than four and 65(30.1%) had four to six family sizes. In addition 15(6.9%), 52(24.1%), 76(35.2%) and 73(33.8%) have estimated monthly income less than 40$, 40$-56$, 57$-73$ and more than 73$ respectively. In considering the mortality, 61.1% of the neonates were died among families having family size more than six and 44.4% were among the families having estimated monthly income of less than 40$.

Most of the mothers (72.2%) were urban residents and around one fifth (19.9%) have a distance more than 50 kilometers between their home and the nearby hospital. Similarly, 43(19.9%) of them have between 10–50 kilometers and 130(60.2%) had less than 10 kilometers between their home and the nearby hospital. In considering the maternal habits, 23(10.6%) of the mothers and 27(12.5%) of the mothers had a habit of chewing khat and alcohol intake respectively **Table 1**).

**Table 1:** Socio-demographic characteristics of neonates with low birth weight and their mothers at NICU of public hospitals in Ethiopia.

**Variables**	**Category**	**Total (%)**	**Status of the newborn**		**P value**
			Died [n(%)]	Survived [n(%)]	
Marital status	Single	21(9.7%)	14(77.8%)	7(3.5%)	0.0001
	Married	195(90.3%)	4(22.2%)	191(96.5%)	
Religious status	Orthodox	94(43.5%)	6(33.3%)	88(44.4%)	0.539
	Muslim	41(18.9%)	3(16.7%)	38(19.2%)	
	Protestant	70(32.5%)	7(38.9%)	63(31.8%)	
	Others	11(5.1%)	2(11.1%)	9(4.5%)	
Educational status of the mother	Unable to read and write	28(12.9%)	8(44.4%)	20(10.1%)	0.094
	Read and write	52(24.1%)	5(27.8%)	47(23.7%)	
	Grade 1–8	66(30.6%)	3(16.7%)	63(31.8%)	
	Grade 9–12	44(20.4%)	1(5.6%)	43(21.7%)	
	College and above	26(12.0%)	1(5.0%)	25(12.6%)	
Maternal occupational status	House wife	22(10.2%)	7(38.9%)	15(7.6%)	0.431
	Self-employee	55(25.5%)	6(33.3%)	49(24.7%)	
	Farmer	67(31.0%)	3(16.7%)	64(32.3%)	
	Merchant	49(22.7%)	1(5.6%)	48(24.2%)	
	Civil servant	23(10.6%)	1(5.6%)	22(11.1%)	
Family size	Less than four	101(46.8%)	2(11.1%)	99(50.0%)	0.0001
	4–6	65(30.1%)	5(27.8%)	60(30.3%)	
	More than 6	50(23.1%)	11(61.1%)	39(19.7%)	
Estimated monthly income	<40$	15(6.9%)	8(44.4%)	7(3.5%)	0.213
	40$-56$	52(24.1)	5(27.8%)	47(23.7%)	
	57$-73$	76(35.2%)	3(16.7%)	73(36.9%)	
	>73$	73(33.8%)	2(11.1%)	71(35.9%)	
Place of Residence	Urban	156(72.2%)	3(16.7%)	153(77.3%)	0.0001
	Rural	60(27.8%)	15(83.3%)	45(22.7%)	
Distance b/n home and hospital(in Km)	Less than 10	130(60.2%)	2(11.1%)	128(64.6%)	0.401
	10–50	43(19.9%)	5(27.8%)	38(19.2%)	
	More than 50	44(20.4%)	11(61.1%)	32(16.2%)	
Maternal habit of alcohol intake	Yes	27(12.5%)	11(61.1%)	16(8.1%)	0.0001
	No	189(87.5%)	7(38.9%)	182(91.9%)	
Maternal habit of chewing khat	Yes	33(15.3%)	12(66.7%)	11(56%)	0.0001
	No	193(89.4%)	6(33.3%)	187(94.4%)	

### Maternal medical and obstetrical factors

Regarding to the number of antenatal care visits, 24(11.1%) of the mothers did not attend any antenatal care. The remaining 66(30.6%), 49(22.7&), 47(21.8%) and 30(13.9%) of the mothers attend one, two, three and four and above antenatal visits respectively. In considering mortality among the dyed newborns, maximum number (33.3%) of the dyed were among mothers who did not attend any antenatal visits while the remaining 5(27.8%), 3(16.7%), 2(11.1%) and 2(11.1%) were among the categories of mothers who have attended one, two, three and four & above antenatal visits respectively. In considering total number of pregnancy, 74(34.3%), 44(20.4%) and 98(45.4%) of the mothers were primigravid, two to four and above four instance of pregnancy respectively. Seventy four (34.3%), 44(20.4%) and 98(45.%) of the mothers had one, two to four and above four number of alive births respectively and among them 8(44.4%), 6(33.3%) and 4(22.2%) of the newborns were dyed among each categories of parity respectively.

Nearly three fourth (75.5%) of the neonates were delivered at health institution and 102(47.2%) of the new born delivery was attended by health professionals and the remaining 61(28.2%), 26(12%) and 27(12.5%) were attended by health extension workers, trained traditional birth attendants and relatives respectively. Magnitude of mortality was high (66.7%) among the home delivered newborns and least number (11.1%) of neonates were died among delivery attended by health professionals. Regarding to the gestational age of the newborn, 89(41.2%), 126(58.3%) and 1(0.5%) of the newborns were delivered at less than 36, 37–72 and more than 42 weeks of gestation respectively and among each categories of gestational age, 10(55.6%), 7(38.9%) and1(5.6%) died respectively.

Regarding the medical history of the mothers, 57(26.4%), 55(25.5%), 37(17.1%), 51(23.6%), 19(8.8%) and 22(10.2%) of the mothers have a history of pregnancy induced hypertension, bleeding during pregnancy, urinary tract infection or sexually transmitted infection, iron deficiency anemia, diabetes mellitus HIV/AIDS respectively (**Table 2**).

**Table 2:** Maternal medical and obstetrical characteristics of neonates with low birth weight and their mothers at NICU of public hospitals in Ethiopia.

**Variables**	**Category**	**Total (%)**	**Status of the newborn**		**P value**
			Died [n(%)]	Survived [n(%)]	
Gravidity	One	74(34.3%)	5(27.8%)	69(34.8%)	0.931
	2–4	44(20.4%)	6(33.3%)	38(19.2%)	
	More than 4	98(45.4%)	7(38.9%)	91(46.0%)	
Place of delivery	Health institution	163(75.5%)	6(33.3%)	157(79.3%)	0.721
	Home	53(24.5%)	12(66.7%)	41(20.7%)	
Birth attendant	Relatives	27(12.5%)	7(38.9%)	20(10.1%)	0.741
	TTBA	26(12.0%)	5(27.8%)	21(10.6%)	
	HEW	61(28.2%)	4(22.2%)	57(28.8%)	
	Health professionals	102(47.2%)	2(11.1%)	100(50.5%)	
Pregnancy induced hypertension	Yes	57(26.4%)	13(72.2%)	44(22.2%)	0.325
	No	159(73.6%)	5(27.8%)	154(77.8%)	
Bleeding during pregnancy	Yes	55(25.5%)	11(61.1%)	44(22.2%)	0.162
	No	161(74.5%)	7(38.9%)	154(77.8%)	
Maternal history of UTI/STI	Yes	37(17.1%)	11(61.1%)	26(1.1%)	0.610
	No	179(82.9%)	7(38.9%)	172(86.9%)	
Intra partum fever	Yes	27(12.5%)	9(50.0%)	18(9.1%)	0.412
	No	189(87.5%)	9(50.0%)	180(90.9%)	
History of infant death	Yes	46(21.3%)	10(55.6%)	36(18.2%)	0.014
	No	170(78.7%)	8(44.4%)	162(81.1%)	
Mode of delivery	Spontaneous vaginal delivery	154(71.3%)	9(50.0%)	145(73.2%)	0.404
	Assisted instrumental	42(19.4%)	8(44.4%)	34(17.2%)	
	Cesarean section	20(9.3%)	1(5.6%)	19(9.6%)	
Gestational age	<37 weeks	131(60.6%)	10(55.6%)	121(61.1%)	0.143
	37–41 weeks	84(38.9%)	7(38.9%)	77(38.9%)	
	≥42 weeks	1(0.5%)	1(5.6%)	0(0.0%)	
Maternal diagnosed HIV infection	Yes	22(10.2%)	10(55.6%)	12(6.1%)	0.0001
	No	194(89.8%)	8(44.4%)	186(93.3%)	
Maternal DM	Yes	19(8.8%)	13(72.2%)	6(3.0%)	0.0001
	No	197(91.2%)	5(27.8%)	192(97.0%)	
Maternal Iron deficiency anemia	Yes	51(23.6%)	14(77.8%)	37(18.7%)	0.0001
	No	165(76.4%)	4(22.2%)	161(81.3%)	

UTI: Urinary tract infection, STI: Sexually transmitted infection, DM: Diabetes Mellitus, Gravidity: the total number of time that the women has been pregnant

### Neonatal factors

Among all the neonates with low birth weight admitted to the neonatal intensive care unit, 158(73.1%) were cried immediately at birth. Among the newborns delivered at health institution, 33(15.3%) and 130(60.2%) had first minute APGAR score less than seven and above seven respectively. Similarly, 30(13.9%) and 133(61.6%) had the fifth minute APGAR score less than seven and above seven respectively. Similarly, 14(6.5%) of the newborns were resuscitated at birth.

Regarding the exclusive breast feeding initiation among the admitted newborns with low birth weight, 126(58.3%) were initiated exclusive breast feeding within one hour after delivery and 154(71.3%) feed only breast milk during their ages. Similarly, 165(76.4%) were kept in kangaroo mother care within one hour and 42(19.4%) had concurrent health problems (**Table 3**).

**Table 3:** Neonatal characteristics of neonates with low birth weight and their mothers at NICU of public hospitals in Ethiopia.

**Variables**	**Category**	**Total (%)**	**Status of the newborn**		**P value**
			Died [n(%)]	Survived [n(%)]	
Cry immediately at birth	Yes	158(73.1%)	10(55.6%)	148(74.7%)	0.487
	No	58(26.9%)	8(44.4%)	50(25.3%)	
First minute APGAR score (n = 163)	Less than 7	33(20.2%)	12(66.7%)	21(10.6%)	0.415
	Greater than 7	130(79.8%)	6(33.3%)	124(62.6%)	
Fifth minute APGAR score (n = 163)	Less than 7	30(18.4%)	12(66.7%)	18(9.1%)	0.361
	Greater than 7	133(81.6%)	6(33.3%)	127(64.1%)	
Resuscitated at birth	Yes	14(6.5%)	3(16.7%)	11(5.6%)	0.128
	No	202(93.5%)	15(83.3%)	187(94.4%)	
Kept in KMC in one hour	Yes	165(76.4%)	5(27.8%)	160(80.8%)	0.0001
	No	51(23.6%)	13(72.2%)	38(19.2%)	
Does the neonate initiate EBF within one hour?	Yes	126(58.3%)	2(11.1%)	124(62.6%)	0.0001
	No	90(41.7%)	16(88.9%)	74(37.4%)	
Feeding of the neonate within 28 days	Only breast milk	154(71.3%)	4(22.2%)	150(75.8%)	0.0001
	With additional food	62(28.7%)	14(77.8%)	48(24.2%)	
Concurrent health problems	Yes	42(19.4%)	15(83.3%)	27(13.6%)	0.0001
	No	174(80.6%)	3(16.7%)	171(86.4%)	

KMC: Kangaroo mother care, EBF: Exclusive breast feeding

### The newborns survival status

**Fig 1:**
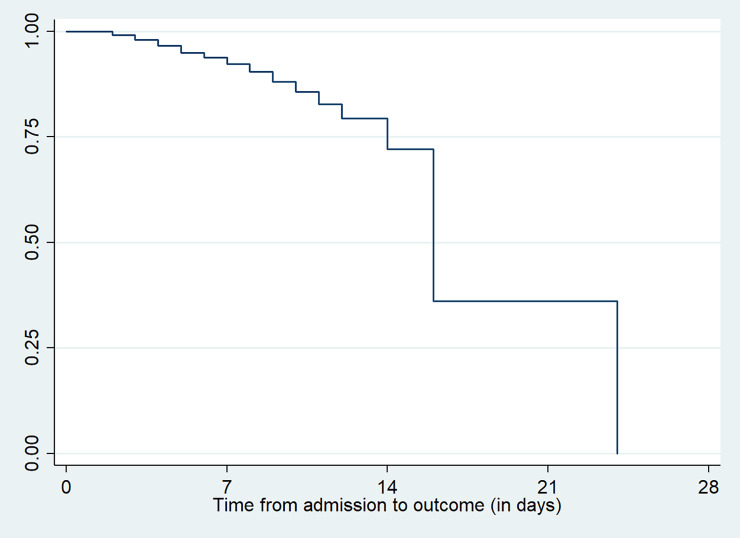
The Kaplan Meier survival estimates among neonates admitted with low birth weight at public hospitals in Ethiopia.

The cumulative proportion of surviving at the end of third days was 99.01% (95%CI: 96.11, 99.75). Similarly it was 97.81% (95%CI: 94.25, 99.18), 96.27% (95%CI: 91.76, 98.33), 94.33% (95%CI: 88.72, 97.19) and 91.46% (95%CI: 83.91, 95.55) at the end of fourth, fifth, sixth and seventh day respectively. The overall mean survival time was 17.13 days (95%CI: 12.76, 21.49) while the overall median survival time was 16 days (95%CI: 13.19, 18.81). There was no any observed death at the first follow up day and there were equal number of death (two deaths) at the second, third, fourth and fifth days. More than one fourths (22.2%) of the deaths were reported within the first three days and 44.4% of the deaths were observed within the first seven days whereas the number of deaths observed within the first 14 days of follow up were 44.8%. In this study, 18(8.3%) of the newborns with low birth weight were died and 198 (91.7%) were recovered. In addition, there was no transfer out, no neonate who withdrew treatment, no any loss to follow up and no any follow up period completed before their status was known (**Fig 1**).

### The neonatal mortality rate among LBW neonates

The mortality rate among neonates admitted with low birth weight was 83 per 1000 live births. In addition, neonates were followed for a total of 1240 days. Hence; the overall incidence density rate of neonatal mortality among newborns admitted with low birth weight was 14.5 per 1000 neonate days (person time of observation).

### The log rank estimate of mortality among the covariates of the variables

Among the socio demographic characteristics, marital status, place of residence, maternal history of chewing khat, maternal history of alcohol intake were variables which have p-value less than 0.05 in log rank estimate of mortality (**Table 4**).

**Table 4:** The log rank estimate of socio-demographic characteristics of the newborn with low birth weight and their mothers.

**Variables**	**Log rank estimate**
Age of the neonate	X^2^ = 0.828, P-value = 0.363
Sex of the neonate	X^2^ = 0.31, P-value = 0.578
Maternal age	X^2^ = 0.918, P-value = 0.61
Marital status	X^2^ = 61.24, P-value = 0.0001
Religious status	X^2^ = 2.16, P-value = 0.539
Educational status of the mother	X^2^ = 7.93, P-value = 0.094
Occupational status of the mother	X^2^ = 0.68, P-Value = 0.431
Family size	X^2^ = 13.90, P-value = 0.001
Estimated monthly income	X^2^ = 3.032, P-value = 0.213
Place of residence	X^2^ = 19.349, P-value = 0.0001
Distance between home and hospital	X^2^ = 1.67, P-value = 0.401
Habit of chewing khat	X^2^ = 54.85, P-value = 0.000
Maternal history of alcohol intake	X^2^ = 30.05, P-value = 0.0001

Among the maternal and obstetrical factors, place of delivery, maternal history of pregnancy induced hypertension, history of maternal iron deficiency anemia, history of maternal diabetes mellitus and history of maternal diagnosed HIV status were variables having p value less than 0.0 in log rank survival estimate (**Table 5**).

**Table 5:** The log rank estimate of maternal medical and obstetrical characteristics of the newborn with low birth weight and their mothers.

**Variables**	**Log rank survival estimate**
ANC visits	X^2^ = 4.23, P-value = 0.376
Gravidity	X^2^ = 0.14, P-value = 0.931
Parity	X^2^ = 2.30, P-value = 0.316
Place of delivery	X^2^ = 0.12, P-value = 0.721
Birth attendants	X^2^ = 1.25, P-value = 0.741
History of pregnancy induced hypertension	X^2^ = 1.92, P-value = 0.325
History of bleeding during pregnancy	X^2^ = 9.80, P-value = 0.162
History of UTI/STI	X^2^ = 2.09, P-value = 0.610
History of maternal iron deficiency anemia	X^2^ = 27.75, P-value = 0.0001
History of maternal DM	X^2^ = 63.61, P-value = 0.0001
Maternal diagnosed HIV	X^2^ = 42.44, P-value = 0.0001
History of infant death	X^2^ = 6.05, P-value = 0.014
Mode of delivery	X^2^ = 1.81, P-value = 0.404
Gestational age	X^2^ = 4.88, P-value = 0.143

**Fig 2:**
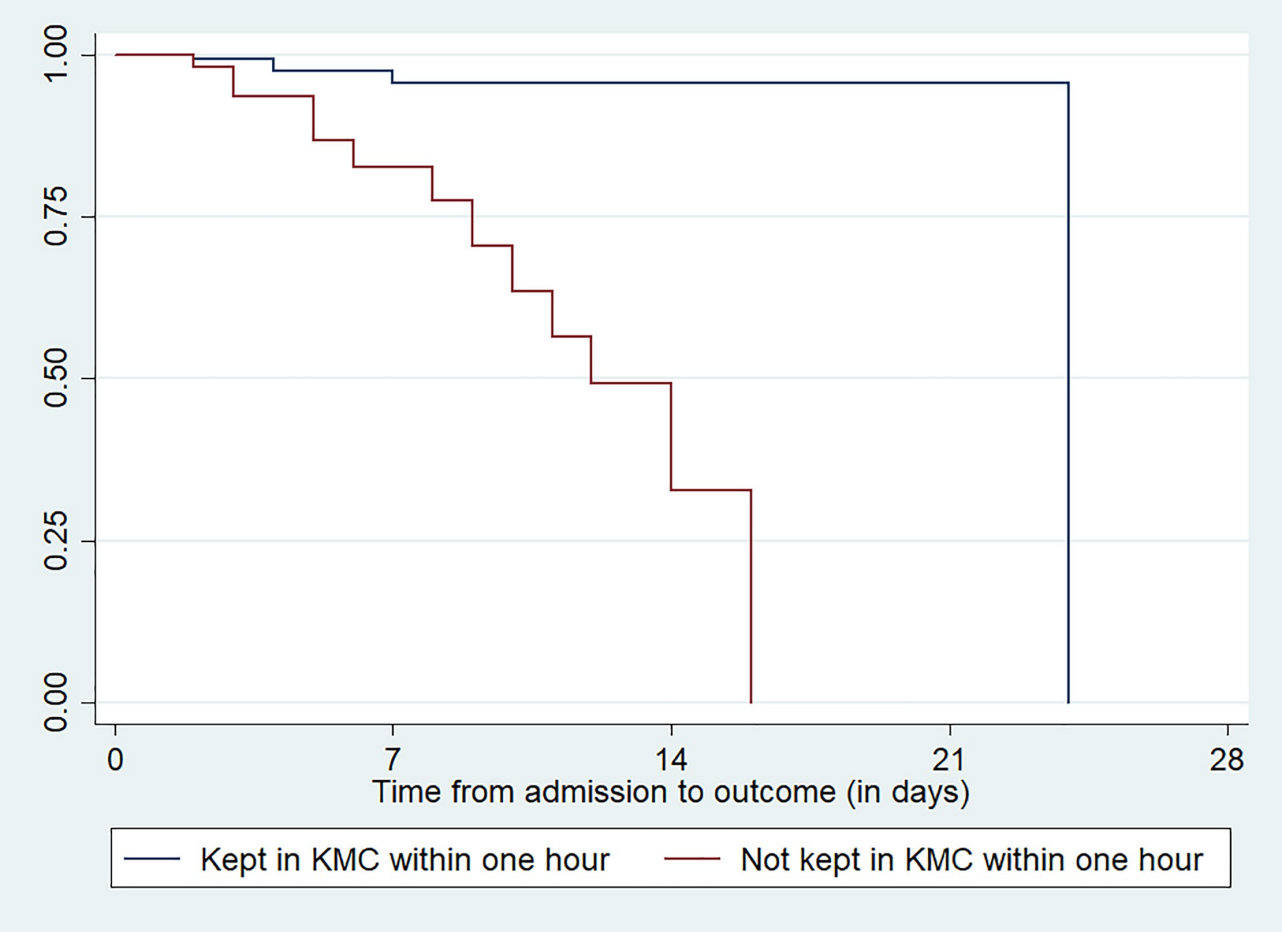
Survival estimation of neonates admitted with low birth weight for the variable kept the neonates in KMC within one hour at public hospitals in Ethiopia.

**Fig 3:**
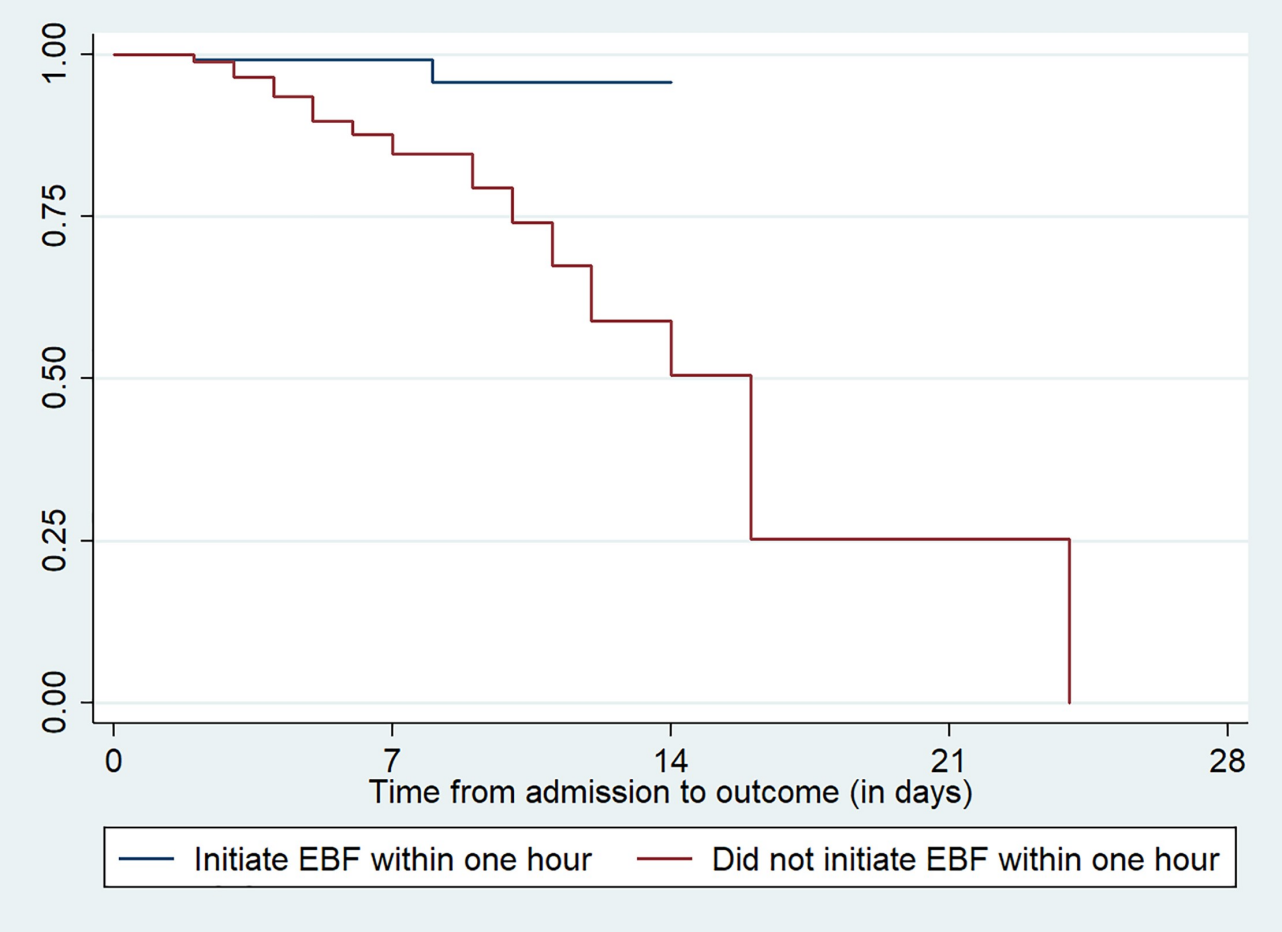
Survival estimation of neonates admitted with low birth weight for the variable exclusive breast feeding initiation within one hour at public hospitals in Ethiopia.

In considering variables related to the neonatal characteristics, neonates kept in kangaroo mother care within one hour, exclusive breast feeding initiation within the first hour of delivery, type of feeding within the first 28 days of life and having history of concurrent health problems were factors that have p-value less than 0.05 in log rank estimate of mortality (**Table 6**). Figs 2 & 3 indicates the survival time for the covariates of kept in kangaroo mother care within one hour and exclusive breast feeding initiation within one hour after delivery among the newborns (**Figs 2 &** 3).

**Table 6:** The log rank estimate of neonatal characteristics of the newborn with low birth weight and their mothers.

**Variables**	**Log rank estimate**
Cry immediately at birth	X^2^ = 0.48, P-value = 0.487
First minute APGAR score	X^2^ = 3.551, P-value = 0.415
Fifth minute APGAR score	X^2^ = 3.848, P-value = 0.361
Resuscitation at birth	X^2^ = 2.31, P-value = 0.128
Kept in KMC within one hour	X^2^ = 20.64, P-value = 0.0001
Feeding of the neonate within 28 days	X^2^ = 14.76, P-value = 0.0001
Does the neonate initiate exclusive breast feeding within one hour	X^2^ = 12.45, P-value = 0.0001
Concurrent health problems	X^2^ = 28.35, P-value = 0.0001

### The Kaplan Meier estimates of mean survival time

In this study, since the maximum observation time was censored, the median survival time was not determined; hence the mean survival time was the best measure of central tendency. The overall mean survival time for the predictor maternal history of diabetes maternal history of having HIV infection mellitus, keeping the newborn in kangaroo mother care within one hour of delivery and initiating exclusive breast feeding within one hour was 17.13: 95%CI: 12.76, 21.49) days (**Table 7**).

**Table 7:** The Kaplan Meier estimates of mean survival time for the covariates of variables among the newborns admitted at neonatal intensive care unit in public hospitals in Ethiopia.

**Variables**	**Category**	**Mean survival time (95%CI)**
Marital status	Single	9.22(5.89, 12.55
	Married	13.61(13.23, 13.99)
Family size	<4	13.69(13.26, 14.12)
	4–6	14.43(12.85, 16.01)
	>6	13.18(8.54, 17.83)
Place of residence	Urban	13(73(13.27, 14.18)
	Rural	14.10(10.04, 18.16)
Habit of chewing khat	Yes	8.93(4.54, 13.32)
	No	15.21(14.49, 15.94)
Maternal history of alcohol intake	Yes	12(90(7.54, 18.26)
	No	15.0(14.22, 15.81)
History of maternal iron deficiency anemia	Yes	13.03(8.75, 17.32)
	No	13.59(13.09, 14.10)
History of maternal DM	Yes	8.74(4.59, 12.90)
	No	15.3(14.49, 16.01)
Maternal diagnosed HIV	Yes	9.76(3.45, 16.06)
	No	14.97(14.20, 15.74)
Kept in KMC within one hour	Yes	23.15(22.18, 24.12)
	No	11.69(9.86, 13.53)
Feeding of the neonate within 28 days	Only breast milk	13.61(13.08, 14.13)
	With additional food	14.15(10.10, 18.19)
Does the neonate initiate exclusive breast feeding within one hour	Yes	13.69(13.26, 14.14)
	No	14.78(11.02, 18.53)
Concurrent health problems	Yes	12.41(9.05, 15.76)
	No	13.74(13.44, 14.04)

### Predictors of mortality

In this study maternal history of diabetes mellitus, maternal history of HIV/AIDS, keeping the newborn in kangaroo mother care within one hour and breast feeding initiation within one hour were statistically significant in multivariable cox regression analysis.

Newborns with low birth weight who have developed by mothers with history of diabetes mellitus have four times higher hazard of mortality as compared with the counterparts who did not have history of diabetes mellitus (AHR: 4.79, 95%CI: 1.15, 19.89). The risk of mortality among newborns with low birth weight delivered by mothers with a history of HIV/AIDS was 6 times higher as compared with mothers who did not have a history of HIV/AIDS (AHR: 6.47; 95%CI: 1.43, 29.34). Newborns with low birth weight who did not kept in kangaroo mother care within one hour of delivery had 13 times higher hazard of mortality as compared with the counterparts who were kept in kangaroo mother care within one hour (AHR: 13.0; 95%CI: 3.42, 49.54). The risk of mortality among newborns with low birth weight who have initiate exclusive breast feeding within one hour was 81% higher as compared with those who did not initiate exclusive breast feeding within one hour (AHR: 0.19; 95%CI: 0.04, 0.95)(**Table 8**).

**Table 8:** Predictors of mortality among newborns with low birth weight in neonatal intensive care unit at public hospitals in Ethiopia.

**Variables**	**Categories**	**Total**	**Neonatal death (%)]**	**CHR(95%CI)**	**AHR(95%CI)**
Marital status	Single	21	66.7%	23.58(7.54, 73.73)	2.59(0.64, 10.43)
	Married	195	2.1%	1	1
Family size	<4	101	1.9%	1	1
	4–6	65	7.7%	0.099(0.02, 0.45)	0.57(0.05, 6.58)
	>6	50	22.0%	0.34(0.117, 1.02)	1.31(0.09, 17.71)
Place of residence	Urban	156	1.9%	1	1
	Rural	60	25.0%	9.92(2.82, 34.92)	0.41(0.11, 1.65)
Maternal habit of chewing khat	Yes	23	52.2%	18.34(6.64, 50.6)	7.19(0.48, 107.1)
	No	193	3.1%	1	1
Maternal habit of alcohol intake	Yes	27	40.7%	9.94(3.71, 26.65)	2.44(0.24, 25.24)
	No	189	3.7%	1	1
History of maternal iron deficiency anemia	Yes	51	27.5%	11.4(3.66, 35.59)	0.38(0.04, 3.24)
	No	165	2.4%	1	1
History of maternal DM	Yes	19	68.4%	22.7(7.89, 65.55)	4.79(1.15, 19.8)*
	No	197	2.5%	1	1
Maternal diagnosed HIV	Yes	22	45.5%	14.67(5.38, 39.9)	6.47(1.43, 29.3)*
	No	194	4.1%	1	1
Kept in KMC within one hour	Yes	165	3.0%	1	1
	No	51	25.5%	8.8(2.85, 27.15)	13.0(3.42, 49.5)*
Feeding of the neonate within 28 days	Only breast milk	154	2.6%	1	1
	With additional food	62	22.6%	6.84(2.19, 21.29)	0.73(0.11, 4.67)
Does the neonate initiate exclusive breast feeding within one hour	Yes	126	1.6%	1	0.19(0.04, 0.95)*
	No	90	17.8%	9.12(2.06, 40.31)	1
Concurrent health problems	Yes	42	35.7%	3.73(1.98, 7.01)	0.69(0.13, 3.62)
	No	174	1.7%	1	1

*Variables which have p-value <0.05 in multivariable cox regression analysis

## Discussion

This study determines the Kaplan Meier estimates of mortality and its predictors among newborns admitted with low birth weight at neonatal intensive care unit at public hospitals in Ethiopia. The risk of mortality among newborns with low birth weight was high at the first two weeks of follow up and decreases while the follow up time was increased. This study finding was consistent with other previously conducted studies at England [15–17]. In addition; this might be due to the delay to the initiation of treatment for those neonates delivered at health center or home, where they were prone to certain health problems or complications because the survival status among neonates who initiate treatment immediately after delivery and the later ages.

The incidence of mortality among newborns admitted with low birth weight was 83 per 1000 live births which were higher than the study conducted at in two health districts of Burkina Faso and estimated national mortality rate which was 28 per 1000 live births [18, 19]. This study finding is less than the study conducted at rural Ethiopia, where the incidence rate of mortality was 110 per 1000 live births and the study conducted at Bangladesh which was 133 per 1000 live births [20, 21].

This might be due to the variation in difference in implementation strategy among the local health offices administrators and area in residence of the populations. In addition; this might be due to inadequate healthcare seeking habit of the newborn mothers or caretakers and/or families for neonatal and infancy and childhood health problems in such institutions [22, 23]. It might also be related with the low accessibility or availability and reduced quality of health care supplies at the study setting [24, 25].

Newborns with low birth weight who have developed by mothers with history of diabetes mellitus have four times higher hazard of mortality as compared with the counterparts who did not have history of diabetes mellitus (AHR: 4.79, 95%CI: 1.15, 19.89). This study finding is consistent with the study conducted at rural Ethiopia [20]. This might be related to the complications occurred due to diabetes mellitus during pregnancy because diabetes mellitus can result in hypoglycemia, hypocalcaemia, respiratory distress, growth restriction, polycythaemia, increased magnesium amount, respiratory distress, congenital anomalies, and excessively increased amount of bilirubin [26–28].

Consistent with the study conducted at University of Gonder specialized hospital, the risk of mortality among newborns with low birth weight delivered by mothers with a history of HIV/AIDS was 6 times higher as compared with mothers who did not have a history of HIV/AIDS (AHR: 6.47; 95%CI: 1.43, 29.34) [29]. This might be due to the maternal immune-compromization, which limits the mother from breast feeding and increased burden of medical costs because the newborns with low birth weight delivered with HIV infected mothers can cost 75% increased costs than those newborns with low birth weight delivered from HIV free mothers [30].

Similar with the study conducted at Palwal and Firdabad districts in the state of Haryana, India, newborns with low birth weight who did not kept under kangaroo mother care within one hour of delivery had 13 times higher hazard of mortality as compared with the counterparts who were kept under kangaroo mother care within one hour (AHR: 13.0; 95%CI: 3.42, 49.54) [31]. This might be due to kangaroo mother care enhances early and continuous skin-to-skin contact between mother and baby as well as promotes exclusive breastfeeding [32].

Evidence derived from hospital-based studies shows that kangaroo mother care results in a forty percent relative reduction in mortality, a fifty eight percent relative reduction in the risk of hospital acquired infections or sepsis, short duration of hospital admission, and a lower risk of reduced respiratory tract infections. In addition; it increases babies’ needs of warmth, increased breast feeding, and protects the baby from infection, safety, and love [33]. Similarly prolonged skin to skin contact has enhances effective body temperature regulation for preterm babies and newborns having low birth weight [31, 33].

The risk of mortality among newborns with low birth weight who had initiate exclusive breast feeding within one hour was 81% higher as compared with those who did not initiate exclusive breast feeding within one hour (AHR: 0.19; 95%CI: 0.04, 0.95). This study finding was consistent with the study conducted at Ghana [34]. This could be commonly associated with the fact that breast milk acquires all the required nutrients for an infant in the first six months of life and it protects the newborn from diarrhea and common childhood health problems or illnesses [35]. In addition; early initiation of exclusive breast feeding enables the newborn to receive the colostrum or first milk, which is full of protective factors [36]. Early initiation of exclusive breast feeding prevents nearly twenty percent of the new born death and thirteen percent of under-five mortality [37].

The first minute and the fifth minute APGAR score, gestational age, multiple gestation, concurrent health problems and feeding of the neonate within the first 28 days of life were declared as statistically significant in previous studies but they are not statistically significant in this study [21, 38–40]. Therefore; meta-analysis study is required.

## Conclusion

The survival status of the neonates admitted with low birth weight was low at the early admission period especially at the first week and gradually increases at the later follow up weeks (after two weeks of follow up). The incidence of neonatal mortality was high and maternal history of diabetes mellitus, HIV/AIDS infection, keeping the newborn in kangaroo mother care and exclusive breast feeding initiation were statistically significant in multivariable cox regression analysis.

Neonates admitted to the NICU with LBW should be intensively followed especially at the early admission periods and the practice of keeping the newborn under KMC within one hour should be highly encouraged. In addition; mothers should be informed and encouraged to initiate exclusive breast feeding immediately after birth and neonates delivered with mother having DM and HIV/AIDS should be followed and encouraged to attend the health institution regularly.

## Supporting information

S1 Data (DTA)

S2 Data (DOCX)

## Abbreviations

AHR: Adjusted hazards ratio
ANC: Antenatal Care
APGAR: Appearance pulse Grimace Activity Respiratory effort
CHR: Crude hazards ration
DM: Diabetes mellitus
EBF: Exclusive breast feeding
KMC: Kangaroo mother care
LBW: Low birth weight
NICU: Neonatal intensive care unit
STI: Sexually transmitted infection
UTI: Urinary tract infection
WHO: World Health Organization
