# Supplementary material for: The Kaplan Meier estimates of mortality and its predictors among newborns admitted with low birth weight at public hospitals in Ethiopia
Source: PLoS One. 2020 Sep 11;15(9):e0238629. doi: 10.1371/journal.pone.0238629 (PMC7485805; doi:10.1371/journal.pone.0238629)
Supplement: S2 Data — (DOCX) [file pone.0238629.s002.docx]

## Data collection tool

**Title:** The Kaplan Meier estimates of mortality and its predictors among newborns admitted with low birth weight at public hospitals in Ethiopia

Date of Admission --------------/-----------------/-----------------

Date of study participant was Died -----------/----------/-----------------

Date of study participant lost to follow up -----------/----------/--------

Date of the study participant with draw the treatment_________/_______/________

Date of the study Participant transferred out_________/_________/__________

Total No of days the neonates has been followed___________ Day.

- Guide to data collectors: Read the variables and fill the data extraction form carefully by making appropriate circle or writing the response on the space provided accordingly.

**Part-I SOCIO DEMOGRAPHIC CHARACTERISTICS**

| **S.No** | **Variables** | **Categories** | **Skip** |
| --- | --- | --- | --- |
| 101 | Code |  |  |
| 102 | Age of Neonate | ------------(in Days) |  |
| 103 | Sex of Neonate | 1. Male 2. Female |  |
| 104 | Age of the Mother | __________ |  |
| 105 | Marital status | 1. Single 2. Married 3. Divorced 4. Widowed |  |
| 106 | Religious status | 1. Orthodox 2. Muslim 3. Protestant 4. Others |  |
| 107 | Educational status | 1. Unable to read and write 2. Read and write 3. Grade 1-8 4. Grade 9-12 5. College and above |  |
| 108 | Maternal occupational status | 1. House wife 2. Self-employee 3. Farmer 4. Merchant 5. Civil servant |  |
| 109 | Family size | __________ (in number) |  |
| 110 | Estimated monthly income | __________(in USD) |  |
| 111 | Place of residence | 1. Urban 2. Rural |  |
| 112 | Distance b/n home and hospital(in Km) | __________ |  |
| 113 | Maternal habit of alcohol intake | 1. Yes 2. No |  |
| 113 | Maternal habit of chewing khat | 1. Yes 2. No |  |

**PART-II; Maternal Factors**

| S.No | Variables | **Categories** | Skip |
| --- | --- | --- | --- |
| 201 | Does the mother have ANC f/up for this birth? | 1. Yes 2. No | If 2 go to 203 |
| 202 | How many visits did she attend? | 1. One visit 2. Two visits 3. Three visits 4. Four visits |  |
| 203 | Does the mother have previous history of pregnancy? | 1. Yes 2. No | If 2 go to 301 |
| 204 | How many Total pregnancies does she have (Gravidity)? | __________________ |  |
| 205 | How many alive births does she has (Parity)? | _______________ |  |
| 206 | What was the birth interval b/n the current birth and birth before this birth? | ________ Years |  |
| 207 | Where did the mother delivered? | 1. Health institution 2. Home |  |
| 208 | Who did attend the birth? | 1. Relatives 2. TTBA 3. HEW 4. Health professionals |  |
| 209 | Did the mother have pregnancy induced hypertension? | 1. Yes 2. No |  |
| 210 | Did the mother have bleeding during pregnancy? | 1. Yes 2. No |  |
| 211 | Did the mother have history of UTI/STI? | 1. Yes 2. No |  |
| 212 | Did the mother have intrapartum fever? | 1. Yes 2. No |  |
| 213 | Did the mother have history of infant death? | 1. Yes 2. No |  |
| 214 | What was the mode of delivery? | 1. Spontaneous vaginal delivery 2. Assisted instrumental delivery 3. Cesarean section |  |
| 215 | What was the gestational age of the newborn? | ____________ (in weeks) |  |
| 216 | Did the mother have diagnosed HIV infection? | 1. Yes 2. No |  |
| 217 | Did the mother have diabetes mellitus? | 1. Yes 2. No |  |
| 218 | What was the hemoglobin level of the mother? | ____________ |  |

**PART-III Neonatal Factors**

| **S.No** | **Variables** | **Categories** | Skip |
| --- | --- | --- | --- |
| 301 | Did the newborn cry immediately at birth? | 1. Yes 2. No |  |
| 302 | What is the score of first minute APGAR score? | ____________ |  |
| 303 | What is the score of fifth minute APGAR score? | ------------------- |  |
| 304 | Does the newborn resuscitated at birth? | 1. Yes 2. No |  |
| 305 | Does the newborn kept under KMC within one hour? | 1. Yes 2. No |  |
| 304 | Does the Neonate initiate EBF? | 1. Yes 2. No | If 2 go to 306 |
| 305 | When did the neonate start EBF? | 1. Within 1 hour 2. After 1 hour |  |
| 306 | What was the feeding of the newborn within the first 28 days? | 1. Only breast milk 2. With additional food |  |
| 306 | Does the newborn have concurrent health problems? | 1. Yes 2. No |  |
